# Real-world Studies Link NSAID Use to Improved Overall Lung Cancer Survival

**DOI:** 10.1158/2767-9764.CRC-22-0179

**Published:** 2022-07-06

**Authors:** Jason Roszik, J. Jack Lee, Yi-Hung Wu, Xi Liu, Masanori Kawakami, Jonathan M. Kurie, Anas Belouali, Simina M. Boca, Samir Gupta, Robert A. Beckman, Subha Madhavan, Ethan Dmitrovsky

**Affiliations:** 1 Departments of Genomic Medicine, The University of Texas MD Anderson Cancer Center, Houston, Texas.; 2 Melanoma Medical Oncology, The University of Texas MD Anderson Cancer Center, Houston, Texas.; 3 Biostatistics, The University of Texas MD Anderson Cancer Center, Houston, Texas.; 4 Thoracic/Head and Neck Medical Oncology, The University of Texas MD Anderson Cancer Center, Houston, Texas.; 5 Frederick National Laboratory for Cancer Research, Frederick, Maryland.; 6 Georgetown Lombardi Comprehensive Cancer Center and Innovation Center for Biomedical Informatics, Georgetown University Medical Center, Washington, District of Columbia.; 7 AstraZeneca, Gaithersburg, Maryland.; 8 Cancer Biology The University of Texas MD Anderson Cancer Center, Houston, Texas.

## Abstract

Inflammation is a cancer hallmark. NSAIDs improve overall survival (OS) in certain cancers. Real-world studies explored here whether NSAIDs improve non–small cell lung cancer (NSCLC) OS. Analyses independently interrogated clinical databases from The University of Texas MD Anderson Cancer Center (MDACC cohort, 1987 to 2015; 33,162 NSCLCs and 3,033 NSAID users) and Georgetown-MedStar health system (Georgetown cohort, 2000 to 2019; 4,497 NSCLCs and 1,993 NSAID users). Structured and unstructured clinical data were extracted from electronic health records using natural language processing (NLP). Associations were made between NSAID use and NSCLC prognostic features (tobacco use, gender, race, and body mass index, BMI). NSAIDs were statistically significantly (*P* < 0.0001) associated with increased NSCLC survival (5-year OS 29.7% for NSAID users vs. 13.1% for nonusers) in the MDACC cohort. NSAID users gained 11.6 months over nonusers in 5-year restricted mean survival time. Stratified analysis by stage, histopathology, and multicovariable assessment substantiated benefits. NSAID users were pooled independent of NSAID type and by NSAID type. Landmark analysis excluded immortal time bias. Survival improvements (*P* < 0.0001) were confirmed in the Georgetown cohort. Thus, real-world NSAID usage was independently associated with increased NSCLC survival in the MDACC and Georgetown cohorts. Findings were confirmed by landmark analyses and NSAID type. The OS benefits persisted despite tobacco use and did not depend on gender, race, or BMI (MDACC cohort, *P* < 0.0001). These real-world findings could guide future NSAID lung cancer randomized trials.

**Significance:**

NLP and real-world studies conducted in large cohorts explored whether NSAIDs improved survival across NSCLC stages, histopathology, gender, smoking history, or demographic groups. A statistically significant association between NSAID use and NSCLC survival was found. This provides a rationale for future NSAID randomized NSCLC trials.

## Introduction

Inflammation is a hallmark of cancer. Preclinical, translational, and epidemiologic evidence implicated inflammation as a potential pharmacologic target that improves cancer survival (1–4). NSAIDs inhibit carcinogenesis, but their use in oncology has some toxicities that limit their administration (1–4). Mechanistic and clinical investigations are needed to learn which NSAID-treated cancers exert survival benefits. Real-world population-based studies (5, 6) could prove useful to elucidate whether NSAIDs increase non–small cell lung cancer (NSCLC) survival. Although a real-world study can have some bias and is not an alternative to a randomized clinical trial (7), it can provide a rationale for a practice-changing NSCLC clinical trial.

Real-world studies are important because of the conflicting risk and benefit evidence in the literature on NSAID use, especially for patients with cancer. NSAIDs may shorten the survival of patients in certain cancer types, such as endometrial carcinoma (8). However, NSAIDs improve clinical outcomes in colorectal carcinogenesis, as reviewed previously (1–4). Low-dose aspirin use was recommended for primary prevention of colorectal cancer (9, 10). The NSAID sulindac is active against adenomatous polyposis, a colon cancer–prone syndrome (11). The selective cyclooxygenase-2 (COX-2) inhibitor celecoxib also repressed colorectal adenomatous polyposis (12). COX-2 overexpression can promote tumor growth and suppresses tumor immunity (13).

A randomized clinical trial showed that aspirin prevents colorectal adenomas in patients with prior colorectal cancer (14). Celecoxib prevented sporadic colorectal adenomas, but this increased cardiovascular complications (15). Aspirin decreased colorectal cancer risk when colorectal cancers had increased COX-2 protein expression (14). NSAID use to suppress colon carcinogenesis is tailored to those with favorable risk-benefit profiles (16, 17).

NSAID use decreased gastric cancer risk. Both aspirin and nonaspirin NSAID users had risk reduction (18). Prolonged nonaspirin NSAID use reduced by 25% head and neck cancer incidence risk in a case–control study (19). NSAIDs also improved survival for PIK3CA-altered head and neck cancer (20). Aspirin or NSAID treatment was weakly associated with reduced risk of cutaneous basal cell carcinoma or squamous cell carcinoma (21). The NSAID meloxicam reduced breast cancer metastases in a murine surgical wound model (22). This was consistent with clinical evidence that perioperative NSAID treatment inhibited breast cancer metastasis (23)^.^ A prospective study revealed aspirin use reduced breast cancer risk for women at enhanced familial or genetic risk (24). Together, these studies implicated a therapeutic role for NSAIDs beyond colon carcinogenesis, as reviewed previously (1–4).

NSAID or aspirin studies did not previously provide definitive evidence for reduced lung cancer mortality, but some studies found NSAIDs lowered lung cancer incidence (25–27). A population-based cohort study did not find protection between low-dose aspirin use and lung cancer–specific mortality (28). Yet, ibuprofen treatment reduced the risk of lung cancer mortality in another study while aspirin and acetaminophen use was unassociated with this benefit (29). Added support for a role for COX-2 inhibition in NSCLC was shown in a clinical trial showing biologic activity of celecoxib in the bronchial epithelium of current and former smokers (30). Observations like these indicate the need for larger population-based studies to establish the potential efficacy of NSAIDs in improving overall survival (OS) in NSCLC.

It was hypothesized that NSAIDs augment NSCLC survival. To investigate this possibility, real-world studies were performed including one at MD Anderson Cancer Center (MDACC) that spanned from 1987 through 2015. Real-world studies differ from randomized clinical trials. Randomized trials elucidate efficacy and assess safety in cohorts with strict trial entry criteria that might not reflect the clinical biology of the malignancy in the general population (5, 6). Real-world studies use observational evidence acquired retrospectively or prospectively from electronic health records (EHR) or claims data from large cohorts. They assess safety, efficacy, and events in diverse populations, complementing randomized clinical trials (5, 6).

Real-world studies presented here assess how NSAID use was associated with OS in NSCLC. This study extended prior work that confirmed the therapeutic role of NSAIDs in colorectal cancer (1–4). Natural language processing (NLP) interrogated OS in all examined NSCLC cases, including those with squamous cell cancer (SCC) or adenocarcinoma (AD) histopathology (31, 32). Clinical predictors of lung cancer OS were examined, including stage, body mass index (BMI), smoking history, race, and gender (33–38). Associations between NSAID use and lung cancer OS were evaluated in diverse demographic groups. OS of lung cancer cases treated with or without NSAIDs was independently examined in a separate database from Georgetown-MedStar health system.

Results reported here revealed NSAIDs were statistically-significantly associated with improved NSCLC OS (*P* < 0.0001). Benefits depended on stage and histopathology, but were independent of gender, race, and smoking status. These findings potentially provide a clinically tractable way to increase OS in NSCLC. This study has translational relevance because lung cancer is the most common cause of cancer mortality for women and men (39). This work provides a rationale for future randomized trials to assess the antineoplastic activity of specific NSAIDs in NSCLC.

## Materials and Methods

### MDACC Cohort

Demographic, BMI, clinical, and pathologic data were collected using a modified NLP technique (32) and an MDACC clinical database spanning from November 1987 through 2015. NSAID use and NSCLC survival information were collected from the institutional data warehouse: Federated Information Reporting Environment. NSAID use was documented using NLP. NSAIDs used at the time of the patients’ respective lung cancer diagnoses appear in Supplementary Table S1. The study protocol for lung cancer cases was reviewed by the Institutional Review Board (IRB) and deemed IRB exempted. Retrospective data were not assessed as involving human subjects research. Only deidentified data were used. Protected health information was not collected. OS was defined as the time between the date the patient was first seen with a NSCLC diagnosis and the last contact or deceased date.

### Statistical Analysis of the MDACC Dataset

Study sample sizes were determined by cases in the institutional data warehouse that met selection criteria as in Supplementary Fig. S1 with patient characteristics shown in Table 1A. Statistical analyses evaluated the distribution of the data collected. Frequency tabulations were provided for categorical variables. Mean, SD, range, and quantiles were computed for the continuous variables. Multivariable Cox proportional hazards models tested for NSAID associations were adjusted for age, gender, race, and smoking status. Distributions of NSCLC OS were compared across studied cohorts (based on NSAID use, BMI, or smoking status) using the Kaplan–Meier method and log-rank tests with analyses using R software (40–42). To gauge associations of NSAID use, 5-year NSCLC OS was estimated for NSAID users and nonusers in all and subgroup patients. In addition, the difference of 5-year restricted mean survival time between NSAID users and nonusers was also calculated. Landmark studies were conducted along with covariates analyses to exclude immortal time bias using previously established methods (43). All NSCLC cases were considered as were those with AD or SCC diagnoses. Analyses were for all NSCLC stages and also individual stages I, II, III, and IV cases. For BMI analyses, subgroups were: unhealthy low weight (BMI < 20), healthy (20 ≤ BMI < 25), overweight (25 ≤ BMI < 30), obese (30 ≤ BMI < 35), and morbidly obese (35 ≤ BMI), as in prior work (33, 34).

**TABLE 1A:** Lung cancer patient characteristics from MD Anderson Cancer Center cases

	Lung cancer (*n* = 33,162)	AD (*n* = 14,509)	SCC (*n* = 5,687)
**Age**			
Mean (SD)	61.7 (11.0)	60.9 (11.1)	64.7 (9.93)
Median [Min, Max]	62.0 [8.00, 100]	61.0 [11.0, 98.0]	65.0 [19.0, 100]
**Gender**			
Female	14,865 (44.8%)	7,140 (49.2%)	1,810 (31.8%)
Male	18,297 (55.2%)	7,369 (50.8%)	3,877 (68.2%)
**Race**			
Caucasian/Non-Hispanic White	27,151 (81.9%)	11,681 (80.5%)	4,699 (82.6%)
African American/Black	2,887 (8.7%)	1,172 (8.1%)	580 (10.2%)
Hispanic	1,899 (5.7%)	917 (6.3%)	283 (5.0%)
Others^a^	1,225 (3.7%)	739 (5.1%)	125 (2.2%)
**Smoking status**			
No	4,393 (13.2%)	2,525 (17.4%)	376 (6.6%)
Quit	8,415 (25.4%)	3,581 (24.7%)	1,661 (29.2%)
Yes	8,165 (24.6%)	3,266 (22.5%)	1,596 (28.1%)
Missing	12,189 (36.8%)	5,137 (35.4%)	2,054 (36.1%)
**BMI** **^b^**			
Unhealthy low	1,250 (3.8%)	613 (4.2%)	203 (3.6%)
Healthy	5,031 (15.2%)	2,524 (17.4%)	797 (14.0%)
Overweight	5,116 (15.4%)	2,465 (17.0%)	933 (16.4%)
Obese	2,349 (7.1%)	1,051 (7.2%)	439 (7.7%)
Morbidly obese	1,112 (3.3%)	449 (3.1%)	205 (3.6%)
Missing	18,304 (55.2%)	7,407 (51.1%)	3,110 (54.7%)
**Stage**			
I	1,868 (5.6%)	767 (5.3%)	468 (8.2%)
II	797 (2.4%)	304 (2.1%)	291 (5.1%)
III	3,451 (10.4%)	1,312 (9.0%)	1,066 (18.7%)
IV	5,948 (17.9%)	3,042 (21.0%)	704 (12.4%)
Missing	21,098 (63.6%)	9,084 (62.6%)	3,158 (55.5%)
**NSAID**			
No	30,129 (90.9%)	13,096 (90.3%)	5,024 (88.3%)
Yes	3,033 (9.1%)	1,413 (9.7%)	663 (11.7%)

^a^Others include: American Indian/Alaska Native, Asian, Native Hawaiian/Pacific Islander, Other, and Unknown.

^b^BMI categories: unhealthy low weight (BMI < 20), healthy (20 ≤ BMI < 25), overweight (25 ≤ BMI < 30), obese (30 ≤ BMI < 35), and morbidly obese (35 ≤ BMI).

### Georgetown Cohort

The Georgetown cohort included all patients with NSCLC with NSAID treatment information after diagnosis available in the EHR at Georgetown University Hospital and Washington Hospital Center obtained for cases between January 2000 and May 2019. This study protocol was approved by the Georgetown IRB. The need for informed consent was waived by the IRB. International Classification of Disease (ICD) codes ICD-9/ICD-10 identified the study population from the database. Exclusion criteria included patients with no medication information available after diagnosis, no diagnosis date, and patients without a last follow-up date. At NSCLC diagnosis gender, age, race, BMI, smoking history, medication, and clinical notes were obtained.

BMI was available from the MDACC cohort. Seventeen cases with BMI < 10 kg/m^2^ and three with BMI > 60 kg/m^2^ were excluded from analyses because these values were considered clinically unreliable. ICD-O-3 codes from https://seer.cancer.gov/icd-o-3/ determined cases with pulmonary AD or SCC. OS from the date of NSCLC diagnosis was calculated using the last follow-up or death date*.* The clinical characteristics of the Georgetown cohort appear in Supplementary Table S2.

### Georgetown Cohort Medication Exposure

NSAID exposure was defined as NSAIDs (Supplementary Table S1) used daily or weekly at the time of diagnosis. Data from the dates of diagnosis until last follow-up or end of the study period were collected through retrospective medical record review. NLP of clinical provider notes complemented NSAID medication intake information from structured EHR data. CLAMP (version 1.6.1; ref. 32) extracted all medications and associated attributes (when available) including frequency, route, and NSAID type obtained from the clinical record. A curated list of NSAID medications filtered out non-NSAID medications extracted by CLAMP. Filtered NSAID intake information was verified and curated manually.

### Georgetown Cohort Statistical Analyses

Descriptive statistics summarized the distribution of collected data (Supplementary Table S2) for all patients with examined lung cancer and for AD or SCC cases. Frequency tabulations were provided for categorical variables; median values were displayed for the continuous variable of age. Kaplan–Meier plots and respective log-rank tests assessed individual associations between different variables and OS using the ggsurvplot function in the survminer R package (42). Multicovariable survival analyses were with Cox proportional hazards models and the coxph function in the survival package in R (40, 41). Landmark studies were conducted as was done for the MD Anderson cohort.

### Data Availability

The deidentified clinical data and code for analysis will be available upon request to the corresponding author.

## Results

### Patient Characteristics and NSAID Use: Association with Lung Cancer Survival

Patient characteristics included age, gender, race, smoking status, BMI, stage, and NSAID usage. Table 1A displays 33,162 patients with lung cancer in the MDACC cohort (14,509 with AD and 5,687 with SCC histology). Supplementary Table S2 describes 4,497 NSCLC cases in the Georgetown cohort (1,930 with AD and 879 with SCC). A portion of the cases were without recorded histopathology. Clinical flow diagrams are in Supplementary Fig. S1. The univariable Cox proportional hazards models with variables associated with OS appear in Table 1B.

**TABLE 1B:** Univariable Cox proportional hazards models with variables associated with OS

	Lung cancer				AD				SCC			
Characteristic	*N*	HR	95% CI	*P*	*N*	HR	95% CI	*P*	*N*	HR	95% CI	*P*
**Age**	33,162	1.00	1.00–1.01	<0.001	14,509	1.00	1.00–1.00	0.7	5,687	1.00	1.00–1.01	0.14
**Gender**	33,162				14,509				5,687			
Female		—	—			—	—			—	—	
Male		1.25	1.22–1.28	<0.001		1.24	1.20–1.29	<0.001		1.14	1.07–1.21	<0.001
**Race**	33,162				14,509				5,687			
Caucasian/Non-Hispanic White		—	—			—	—			—	—	
African American/Black		1.18	1.14–1.23	<0.001		1.15	1.08–1.23	<0.001		1.22	1.11–1.34	<0.001
Hispanic		0.96	0.90–1.01	0.10		0.95	0.88–1.03	0.2		1.02	0.89–1.17	0.7
Others		0.80	0.75–0.86	<0.001		0.77	0.70–0.85	<0.001		0.98	0.78–1.23	0.9
**Smoking status**	20,973				9,372				3,633			
No		—	—			—	—			—	—	
Quit		1.29	1.24–1.35	<0.001		1.20	1.13–1.27	<0.001		0.86	0.76–0.97	0.018
Yes		1.32	1.27–1.38	<0.001		1.16	1.09–1.23	<0.001		0.93	0.82–1.06	0.3
**BMI**	14,858				7,102				2,577			
Unhealthy low		—	—			—	—			—	—	
Healthy		0.78	0.73–0.83	<0.001		0.79	0.71–0.87	<0.001		0.75	0.63–0.88	<0.001
Overweight		0.70	0.65–0.74	<0.001		0.69	0.63–0.76	<0.001		0.67	0.57–0.78	<0.001
Obese		0.65	0.61–0.71	<0.001		0.70	0.63–0.78	<0.001		0.55	0.45–0.65	<0.001
Morbidly obese		0.59	0.53–0.64	<0.001		0.70	0.61–0.80	<0.001		0.54	0.44–0.67	<0.001
**Stage**	12,064				5,425				2,529			
I		—	—			—	—			—	—	
II		1.52	1.35–1.70	<0.001		1.56	1.29–1.87	<0.001		1.19	0.99–1.44	0.070
III		3.04	2.82–3.28	<0.001		3.07	2.71–3.48	<0.001		2.43	2.12–2.78	<0.001
IV		6.23	5.79–6.70	<0.001		6.18	5.50–6.95	<0.001		6.04	5.22–6.99	<0.001
**NSAID**	33,162				14,509				5,687			
No		—	—			—	—			—	—	
Yes		0.57	0.55–0.60	<0.001		0.60	0.56–0.64	<0.001		0.58	0.53–0.64	<0.001

NOTE: Patients with missing values were not included in the analysis.

Abbreviations: CI, confidence interval; HR, hazard ratio.

Statistically significant associations were observed in the MDACC cohort for age (*P* < 0.001), men (*P* < 0.001), African Americans/Blacks (*P* < 0.001), those who quit smoking (*P* < 0.001) among other features. These included that a higher BMI was associated with a better OS (*P* < 0.001), more advanced stage was associated with a worse OS (*P* < 0.001) and NSAID usage was associated with an improved OS (*P* < 0.001). Supplementary Table S3 and Supplementary Table S4 displayed multicovariable analysis showing HRs for OS for the MDACC cohort and the Georgetown cohort, respectively. NSAID effects were compared with predictors of clinical lung cancer outcomes: tobacco use, gender, and race (33–38).

**FIGURE 1:**
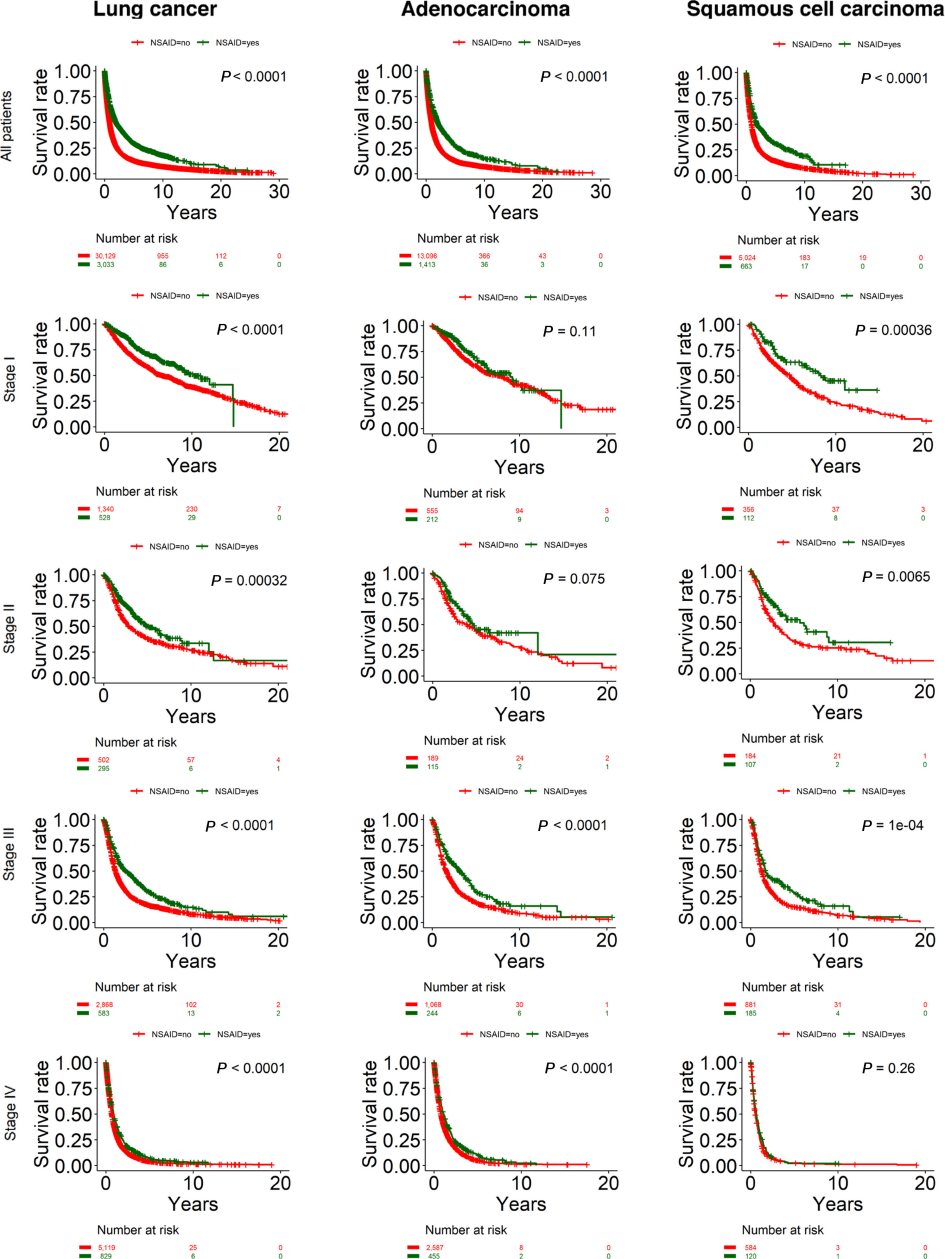
Kaplan–Meier analysis of associated OS and NSAID use in NSCLCs in the MDACC cohort. Kaplan–Meier plots are shown for all as well as stage I, II, III, and IV cases (in rows) for lung cancer, pulmonary AD, and SCC cases (in columns). Red curves represent no NSAID use, while green curves denote NSAID use. Log-rank *P* values are included in each plot.

Substantial associated improvements in NSCLC OS occurred in the MDACC analysis. Kaplan–Meier analyses for all lung cancer cases and individually for stages I, II, III, and IV cases are in Fig. 1. NSAIDs were significantly associated with increased OS (*P* < 0.0001) in all examined lung cancer cases and in AD and SCC cases. Subgroup analyses showed that NSAID protective effects were evident across stages except for stages I and II AD cases and stage IV SCC cases likely due to the relatively small sample sizes of these versus other subsets. The 5-year improved OS rates and the associated gain in 5-year restricted mean survival with NSAID treatments that correspond to Fig. 1 are presented in Supplementary Table S5 for the MD Anderson and Georgetown cohorts. The 5-year OS was 29.7% for NSAID users versus 13.1% for nonusers. NSAID users were associated with a gain of 11.6 months over nonusers in the 5-year restricted mean survival time. The Georgetown cohort (Supplementary Fig. S2) independently confirmed an associated improvement in OS (*P* < 0.0001) in all examined lung cancers and in AD and SCC cases. Increased OS occurred in stage I (AD and SCC), stage III (SCC), and stage IV (AD) NSCLC cases.

To address potential immortal time bias, landmark analyses were performed using multiple landmark times. The obtained results show that NSAID usage is associated with statistically significant longer survivals with landmark times including after 30 days, 180 days, 1 year, 3 years, and 4 years of examination within the MD Anderson Cancer Center dataset (Supplementary Fig. S3). The landmark analysis also showed statistically significant survival associations within the smaller Georgetown cohort with the landmark times 120 days, 180 days, and 1 year (Supplementary Fig. S4). The overall time-dependent Cox model survival was significant (*P* < 0.001 with a HR of 0.87 and a 95% confidence interval of 0.83–0.91).

To determine whether the associated benefit of NSAID usage is independent of NSAID type or associated with specific drugs, we performed a survival analysis with patient groups including patients who used only a single NSAID type, comparing them with nonusers. Significant associations with OS were found for low-dose aspirin (*P* < 0.0001 for the MD Anderson cohort; *P* < 0.0001 for the Georgetown cohort), regular-dose aspirin (*P* < 0.01 for MD Anderson cohort; *P* < 0.01 for Georgetown cohort), celecoxib (*P* < 0.0001 for the MD Anderson cohort; not significant, *n* = 42 for the Georgetown cohort), ibuprofen (*P* < 0.0001 for the MD Anderson cohort; *P* < 0.01 for the Georgetown cohort), and ketorolac (not available for the MD Anderson cohort; *P* < 0.0001 for the Georgetown cohort), as in Supplementary Fig. S5A and S5B. In the MD Anderson Cancer Center cohort, the most active agent was ibuprofen followed by celecoxib, low-dose aspirin, and naproxen in descending order of effectiveness.

### NSAID OS Treatment Benefits: Gender, Race, and Smoking Status

**FIGURE 2:**
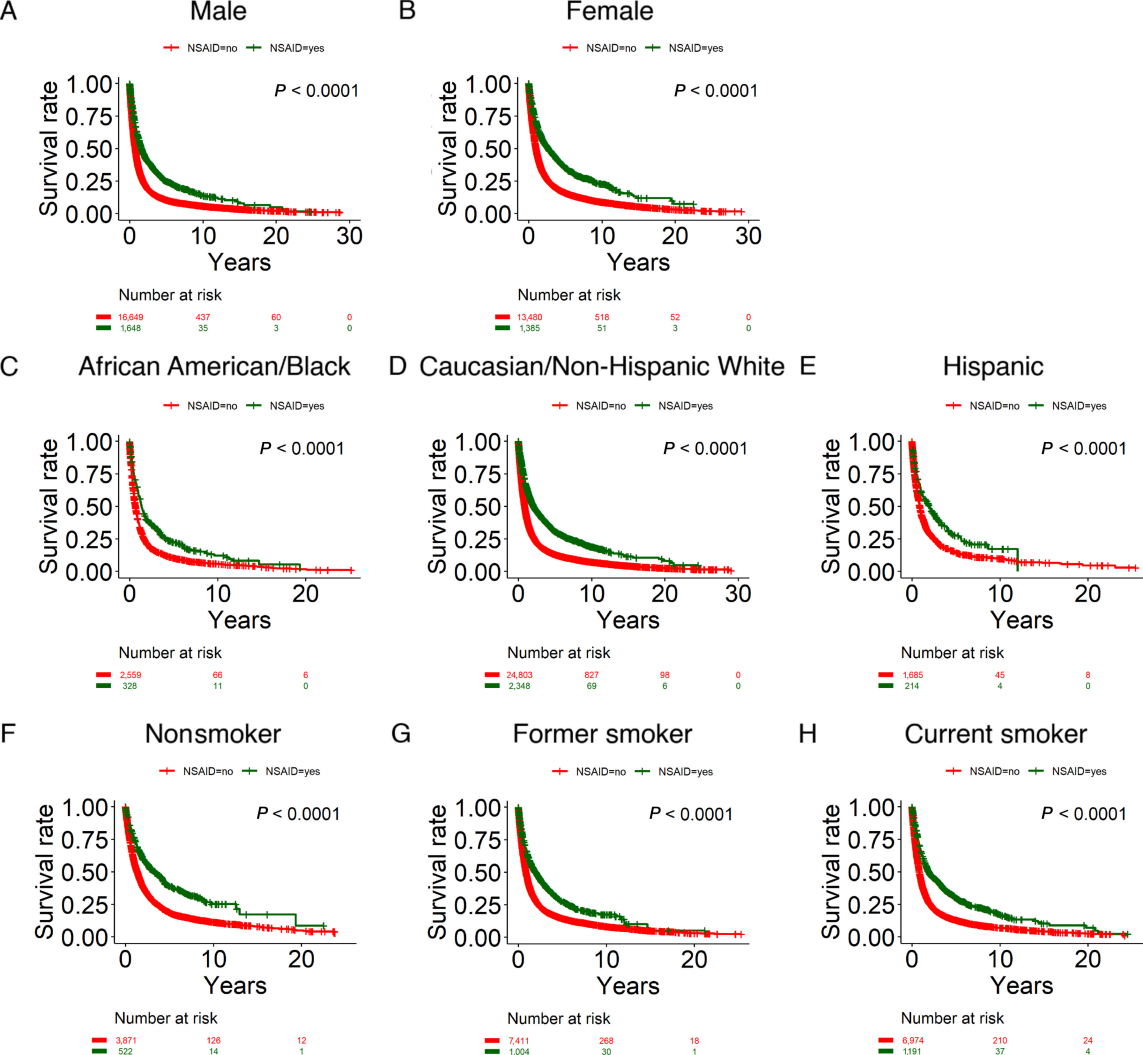
Kaplan–Meier analyses of OS and NSAID use in lung cancer separately examined by gender (**A**: Male, **B**: Female), race (**C**: African American/Black, **D**: Caucasian/Non-Hispanic White, **E**: Hispanic), and smoking status (**F**: Nonsmoker, **G**: Former smoker, and **H**: Current smoker) in the MDACC cohort. Red curves represent no NSAID use, green curves denote NSAID use. Log-rank *P* values are shown in each plot.

**FIGURE 3:**
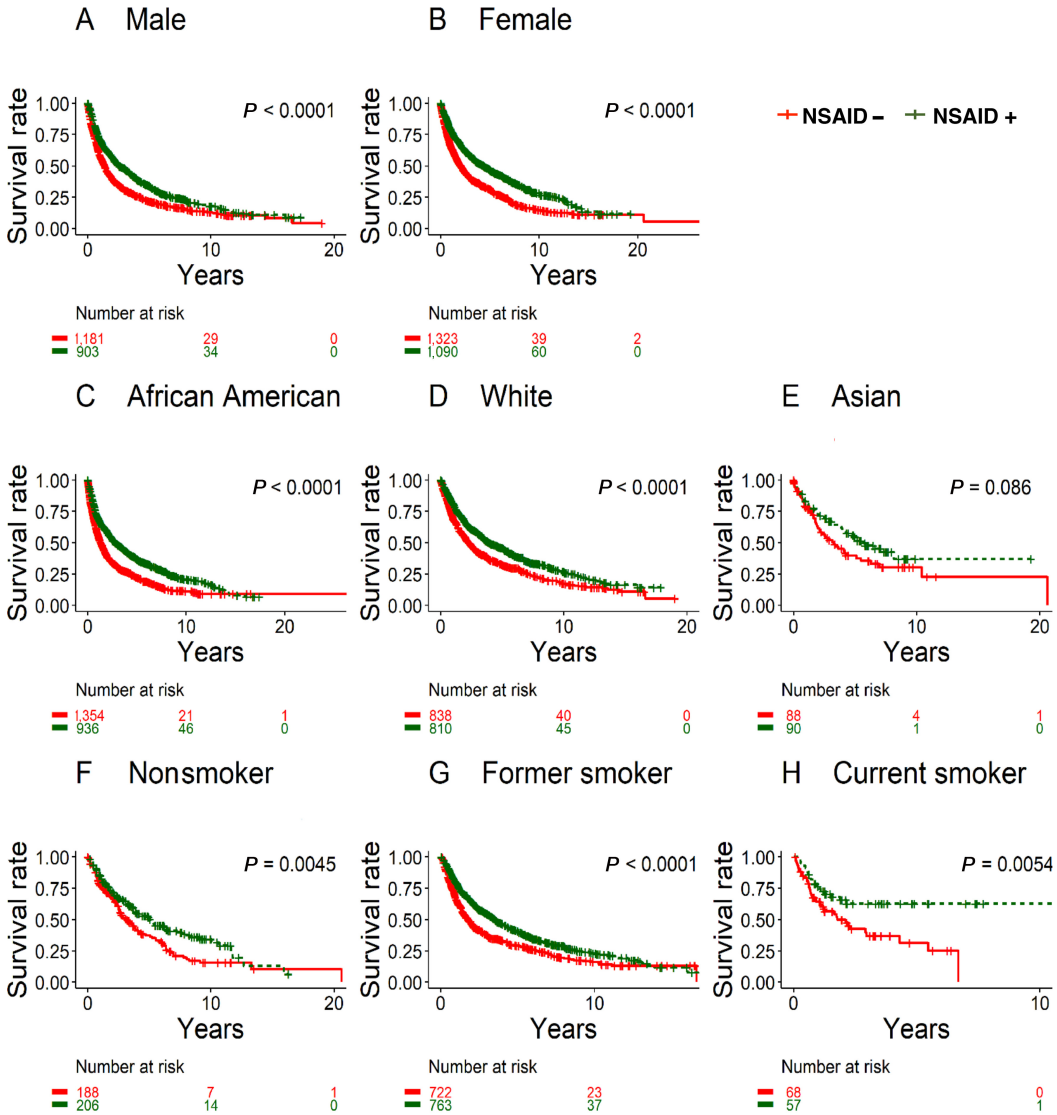
Kaplan–Meier analysis of OS and NSAID use in NSCLCs separately examined by gender (**A**: Male, **B**: Female), race (**C**: African American, **D**: White, **E**: Asian), and smoking status (**F**: Nonsmoker, **G**: Former smoker, and **H**: Current smoker) in the Georgetown cohort. Red curves represent no NSAID (NSAID −) use, green curves denote NSAID (NSAID +) use. Log-rank *P* values are shown in each plot.

NSAID treatments were associated with increased OS in these lung cancer cases independent of gender, race, or smoking status. Kaplan–Meier analysis results in Fig. 2 showed significant (*P* < 0.0001) OS improvements from NSAID use in male and female patients with lung cancer. African American/Black, Caucasian/Non-Hispanic White, and Hispanic NSAID-user patients lived longer than nonusers (Fig. 2, C–E). The MDACC cohort had insufficient numbers of American Indian/Alaska Native, Asian, and Native Hawaiian/Pacific Islanders for robust associations to be made. Stratifications for smoking status showed NSAIDs benefited all studied groups (nonsmokers, former, and current) with significant associated improvements in lung cancer OS (*P* < 0.0001 in Fig. 2F–H). The associated 5-year improved OS rates and the gain in 5-year restricted mean survival with NSAID treatments that correspond to Fig. 2 are presented in Supplementary Table S6. The Georgetown cohort had significant OS associations (*P* < 0.0001) for males, females (Fig. 3A and B), African Americans, and Caucasians/Non-Hispanic/White populations (Fig. 3C and D). There was a favorable trend (*P* = 0.086; Fig. 3E) for those of Asian heritage, but the sample size was insufficient for a significant linkage. Smoking status associations (Fig. 3F–H) were similar to the MDACC cohort.

### BMI and Lung Cancer Survival

**FIGURE 4:**
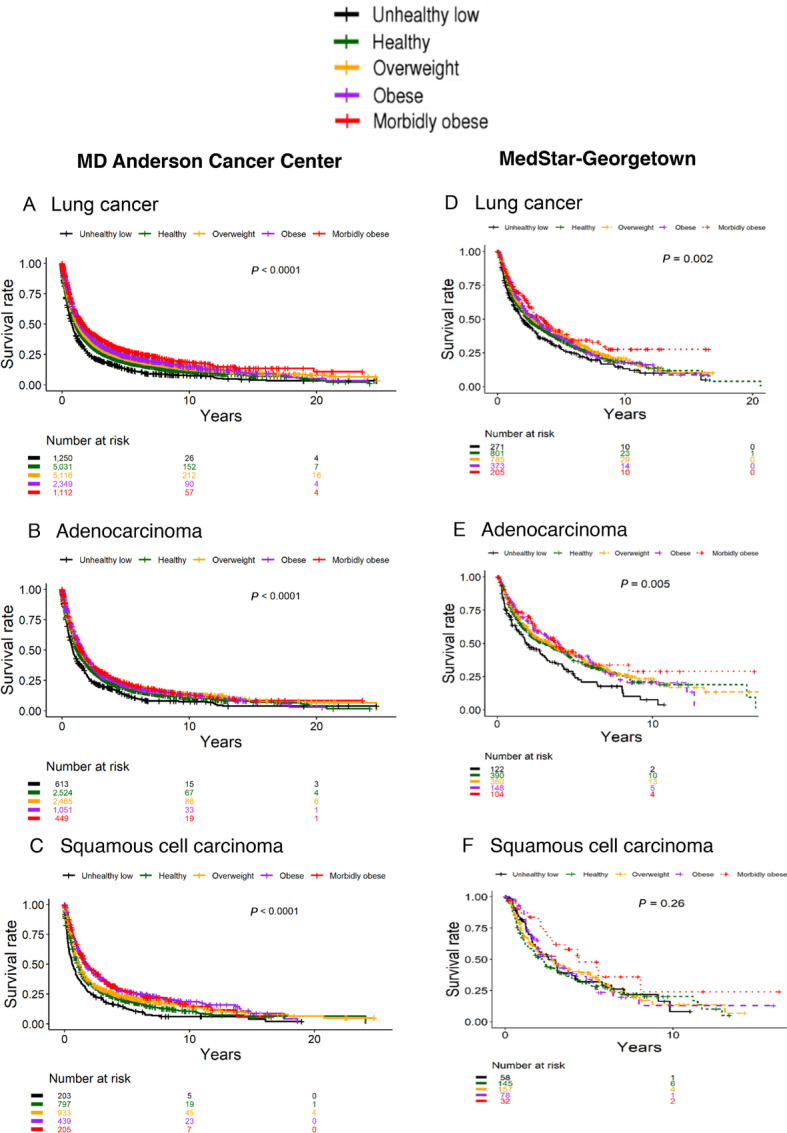
Kaplan–Meier analysis of OS and BMI in NSCLC cases examined in the MD Anderson cohort appear in **A**–**C** while analogous findings from the Georgetown cohort are displayed in **D**–**F**. Kaplan–Meier plots are shown for all NSCLCs, pulmonary AD and SCC, respectively. Independent results from the MD Anderson cohort are displayed in **A** (all lung cancer cases), **B** (AD cases), and **C** (SCC cases), respectively where the black, green, orange, purple, and red curve lines indicate the weight of the patients: unhealthy low (BMI < 20 kg/m^2^), healthy (20 kg/m^2^ ≤ BMI < 25 kg/m^2^), overweight (25 kg/m^2^ ≤ BMI < 30 kg/m^2^), obese (30 kg/m^2^ ≤ BMI < 35 kg/m^2^), and morbidly obese (35 kg/m^2^ ≤ BMI), respectively. Log-rank *P* values are shown in each plot. For the Georgetown cohort (**D**–**F**) black, green, orange, purple, and red curves represent the respective BMI recorded for these examined cases: unhealthy low, BMI < 18.5 kg/m^2^; healthy, 18.5 kg/m^2^ ≤ BMI < 25 kg/m^2^; overweight, 25 kg/m^2^ ≤ BMI < 30 kg/m^2^; obese, 30 kg/m^2^ ≤ BMI < 35 kg/m^2^; morbidly obese, BMI ≥ 35 kg/m^2^, respectively.

The known association between BMI and lung cancer OS (33, 34) was next examined. This independently validated the NLP methodology used in the MD Anderson cohort and Georgetown cohort studies. Statistically significant findings confirmed and extended prior work by finding higher BMI was associated with improved OS in all examined lung cancers, as observed in the MD Anderson cohort with all NSCLC cases (Fig. 4A; *P* < 0.0001) as well as those with AD (Fig. 4B; *P* < 0.0001) and SCC (Fig. 4C; *P* < 0.0001) histopathology. These findings were validated in the Georgetown cohort (Fig. 4D; *P* = 0.002), including those with AD (Fig. 4E; *P* = 0.005), but not those with SCC (Fig. 4F; *P* = 0.26) histopathology. Together, these results implied a possible BMI dose–response relationship in these detected outcome benefits.

### Smoking and OS Lung Cancer in Different Cohorts

**FIGURE 5:**
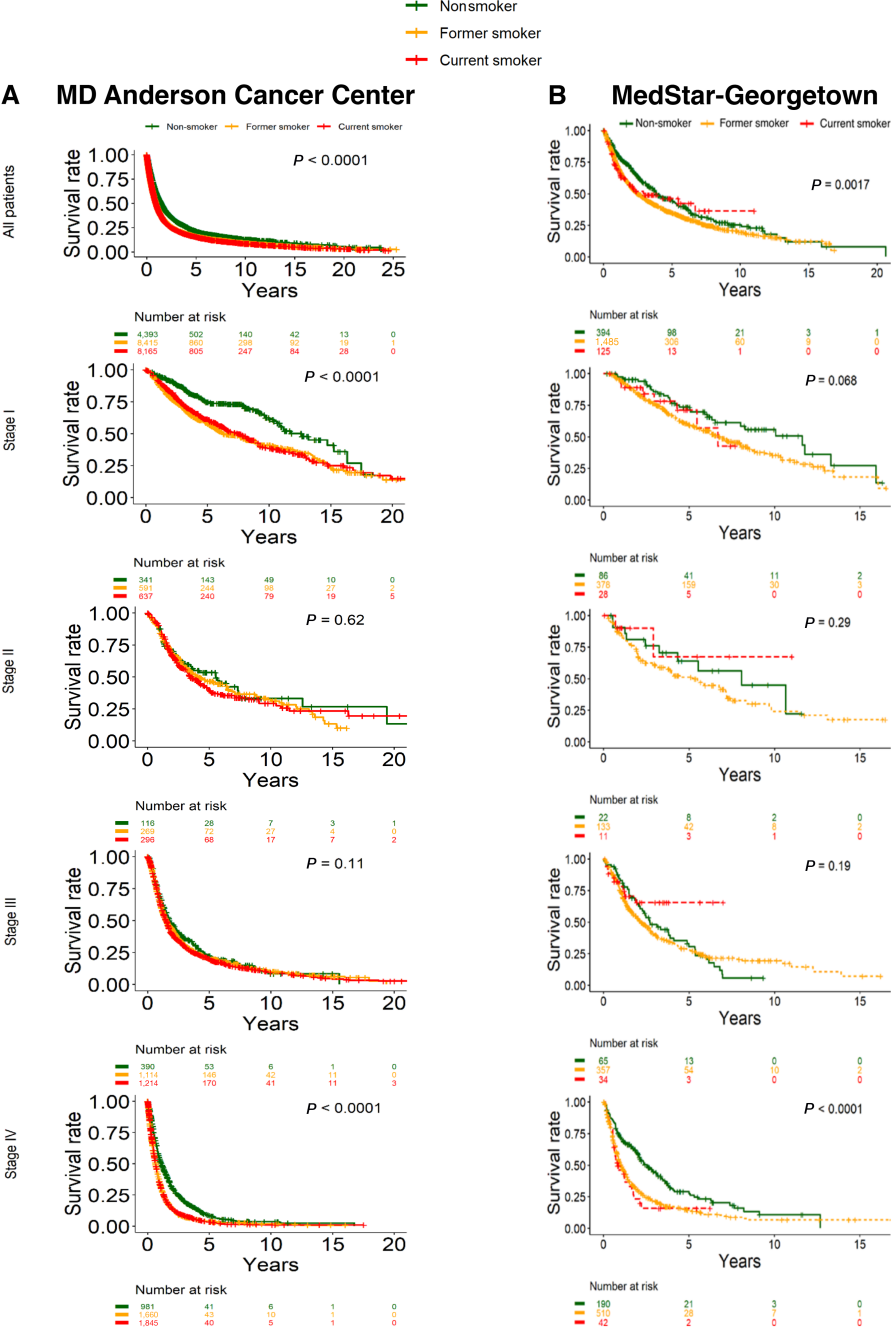
Kaplan–Meier analysis of OS and smoking status in lung cancer. Kaplan–Meier plots are shown for all as well as stage I, II, III, and IV NSCLC cases (in rows) for the MD Anderson Cancer Center (**A**) and Georgetown (**B**) cohorts, respectively (columns). Green, orange, and red curves represent patients who are nonsmokers, former smokers, and current smokers, respectively. Log-rank *P* values are included in the Kaplan–Meier plots.

The next validation compared associations between smoking and lung cancer OS in different populations. Kaplan–Meier analyses in Fig. 5A and B revealed nonsmokers lived longer (MD Anderson cohort: *P* < 0.0001, Georgetown cohort: *P* = 0.0017 in comparisons of all groups) than current or former smokers, as reported in prior work (35, 37). Analyses were made across all stages as well as individually for stage I, stage II, stage III, and stage IV NSCLCs. The associated OS effects of smoking were statistically improved for all stages (*P* < 0.0001) and for stage I (*P* < 0.0001) and stage IV cases (MDACC cohort), as well as for all stages (*P* = 0.0017) with a trend toward improved survival in stage I (*P* = 0.068) and stage IV (*P* < 0.0001) NSCLC cases in the Georgetown cohort.

## Discussion

This comprehensive and real-world (5, 6) study used NLP and population-based approaches to interrogate an MDACC 28-year database to learn whether NSAIDs were associated with improved NSCLC OS. A significant associated benefit occurred in all lung cancers examined and for those with AD and SCC diagnoses. Findings were independently replicated in the Georgetown cohort that spanned 19 years. The robust size and duration of these analyses advance an understanding of how NSAIDs can affect NSCLC survival.

Real-world studies have inherent biases and they are not substitutes for randomized clinical trials (7). One of the main reasons is immortal time bias (43). To address this potential limitation, landmark analyses were performed using multiple landmark times. The obtained results showed highly significant associations with survival in both the MD Anderson and Georgetown cohorts, as in Supplementary Fig. S3 and S4. To investigate the consequences of NSAID treatment in NSCLC, NLP (31) extracted demographic, clinical, and NSAID-use data from these cases at these centers.

Associated improvements in OS occurred across all NSCLCs (men and women) using NSAIDs. Benefits extended to never, former, and current smokers and diverse demographic groups. There was a distinct pattern of associated OS improvement in AD versus SCC subsets of lung cancer. Early SCC and late AD stages of NSCLC cases had significantly prolonged associated OS.

NLP is an informative method to derive insights from unstructured datasets. This is used in translational research (31). Yet, it has some limitations that relate to data fidelity. It is necessary to validate any NLP-based findings. Multiple validations were used here. One was to confirm an improved survival when BMI was assessed in NSCLCs (33, 34). Another validation confirmed that smoking, an unfavorable prognostic factor in lung cancer (35–38), shortened survival in these examined NSCLC cases. An additional approach taken was to establish independently the prognostic contributions of specific clinical characteristics of lung cancer cases such as gender or race. Findings presented here determined the value of such databases to elucidate OS associations in NSCLC. NSAID use was associated with statistically significantly improvements in NSCLC OS. Both AD and SCC NSCLCs benefited. Survival improvements were detected in earlier SCC stages than for AD cases. Hence, NSAID survival improvements were associated with NSCLC stage and histopathology.

Results reported here confirmed that men with NSCLC had statistically higher risk of mortality than women (36). African American/Blacks had a higher lung cancer mortality than Caucasian/Non-Hispanic Whites, as expected from previous work (37). Smoking shortened lung cancer OS, as anticipated from prior work (38). These and other findings that replicated known prognostic factors in lung cancer gave added confidence in the observations presented here.

Improved survival by NSAID use was associated with the NSCLC cases in the MDACC cohort. It was validated in a second, independent, real-world study conducted at Georgetown University Hospital and the Washington Hospital Center, the MedStar-Georgetown cohort. Benefits were detected in all examined NSCLCs, including those from men, women, never, former, and current smokers, independent of race. Benefits also appeared across NSAIDs. The most commonly used NSAIDs, such as low- and regular-dose aspirin, showed similar associated benefits. Ibuprofen was more beneficial in the MD Anderson cohort, but was comparable with aspirin in the Georgetown dataset. Furthermore, ketorolac treatment led to a highly statistically significant associated benefit in the Georgetown cohort, but data on this drug were not available in the MD Anderson dataset. Stage-specific improvements of OS occurred in AD and SCC NSCLCs. In the Georgetown cohort, stage IV AD cases exhibited a significant associated OS improvement. For stage I lung cancers, both cohorts had statistically significant differences detected.

One limitation of this analysis is an inability to assess which NSAID was most efficacious. NSAID dose and treatment length were not determined. Also, NLP for medication extraction could produce low precision or recall (44). Potential associated survival benefits of NSAID use were determined from the medical records. Yet, it was not known whether OS improvements by NSAID users were complicated by clinical toxicities (44).

Another limitation is the precise mechanism that conferred associated survival benefits was not elucidated. One mechanism to consider is *de novo* or posttherapy inflammation after surgery, radiotherapy, immunotherapy, or chemotherapy alone or as part of combined regimens. This could affect lung cancer biology, as reported for breast cancers (22, 23). A murine breast cancer model (recapitulating clinical surgical wounding and inflammation after breast cancer resection) had outgrowth of metastases that were significantly reduced by NSAID use (22). Mechanisms engaged CD8^+^ T cells (22). Surgical wounding increased inflammatory mediators and tumor infiltration by inflammatory macrophages. Perhaps NSAIDs counteracted breast cancer metastasis arising from an unchecked immune response.

The NSAID meloxicam reduced M2 polarization of tumor-infiltrating inflammatory macrophages (22). This antagonized the effects of surgery that altered immune surveillance and allowed outgrowth of disseminated breast cancer cells that would otherwise remain dormant (45–47). A similar mechanism might increase OS in lung cancer after NSAIDs use. Future work should determine whether this or another mechanism is engaged in the associated NSCLC survival improvements that followed NSAID treatments. If survival benefits after NSAID use are confirmed in future randomized clinical trials, this could advance lung cancer practice. This possibility is facilitated by the fact that NSAIDs are relatively inexpensive, cost-effective, and often available without a prescription.

The NSAIDs studied here were largely not selective for COX-2. They inhibited both COX-1 and COX-2 activities. COX-2 selectivity increases cardiovascular complications, especially after chronic use (12). Any future clinical trials using NSAIDs to combat lung cancer should consider COX-2 expression profiles in lung cancers, as reported as important in colorectal adenomas (13, 14).

It is necessary to investigate which NSAID agent is most active in improving OS in lung cancer. Likewise, the precise mechanism(s) through which NSAIDs provide survival benefits should be determined across NSCLC subsets, stages and demographic groups. On the basis of findings reported in breast cancers (22, 47, 48), lung cancer cases benefitting from NSAIDs might exhibit clinical evidence of inflammation at diagnosis.

In addition to systemic effects of NSAIDs, these agents could exert direct effects on lung cancers. Effects might be mediated through differential COX-2 expression because this affects lung cancer prognosis (49). Thus, pathologic and biologic features of NSCLC could help tailor NSAID therapy to specific subsets of lung cancers. Prospective randomized trials should prove useful in the future to address confounding limitations of observational studies like immortal time biases (43, 50). Randomized trials would help establish the therapeutic value of specific NSAIDs in improving survival in lung and potentially other cancers. If activity is confirmed, this would elucidate the precise role played by NSAIDs in lung cancer therapy or prevention.

## Supplementary Material

Supplementary Figure S1 Supplemental Figure 1 displays the studied cohorts at MD Anderson Cancer Center (MD Anderson cohort) and the MedStar-Georgetown University Hospital (Georgetown cohort).

Supplementary Figure S2 Supplemental Figure 2. The Kaplan-Meier analysis of overall survival and NSAID use in lung cancer cases within the MedStar-Georgetown University database (Georgetown cohort).

Supplementary Figure S3 Supplemental Figure 3. Landmark analysis of the MD Anderson Cancer Center lung cancer cases.

Supplementary Figure S4 Supplemental Figure 4. Landmark analysis of the MedStar-Georgetown University Hospital Database (Georgetown cohort).

Supplementary Figure S5 Supplemental Figure 5. The Kaplan-Meier analysis of overall lung cancer survival by NSAID type in (A) the MD Anderson Cancer Center database (MD Anderson cohort) and (B) the Georgetown cohort. Patients who used multiple NSAID types were excluded from this analysis.

Supplementary Table S1 Supplemental Table 1. Nonsteroidal anti-inflammatory drugs analyzed by Natural Language Processing (NLP).

Supplementary Table S2 Supplemental Table 2. Lung cancer patient characteristics in the MedStar-Georgetown University cohort. All lung cancers and those with adenocarcinoma (AD) or squamous cell cancer (SCC) histopathology are shown.

Supplementary Table S3 Supplemental Table 3. Multicovariable Cox proportional hazards model with variables associated with overall survival for the MD Anderson Cancer Center cohort.

Supplementary Table S4 Supplemental Table 4. Multicovariable analysis with variables associated with overall survival for the Georgetown cohort.

Supplementary Table S5 Supplementary Table 5: The MD Anderson and Georgetown cohorts 5-year survival rates and differences of 5-year restricted mean survival time in months between NSAID users and non-users for all patients and by stage and histopathology corresponding to Figure 1. The abbreviations are AD (adenocarcinoma) and SCC (squamous cell cancer), respectively.

Supplementary Table S6 Supplementary Table 6: The MD Anderson cohort 5-year survival rate and difference of 5-year restricted mean survival time in months between NSAID users and non-users by gender, race, and smoking status corresponding to Figure 2. Comparisons are made to the Georgetown cohort.
