# Supplementary material for: Real-world Studies Link NSAID Use to Improved Overall Lung Cancer Survival
Source: Cancer Res Commun. 2022 Jul 6;2(7):590–601. doi: 10.1158/2767-9764.CRC-22-0179 (PMC9273107; doi:10.1158/2767-9764.CRC-22-0179)
Supplement: Supplementary Figure S1 — Supplemental Figure 1 displays the studied cohorts at MD Anderson Cancer Center (MD Anderson cohort) and the MedStar-Georgetown University Hospital (Georgetown cohort). [file crc-22-0179-s01.pptx]

## Slide 1
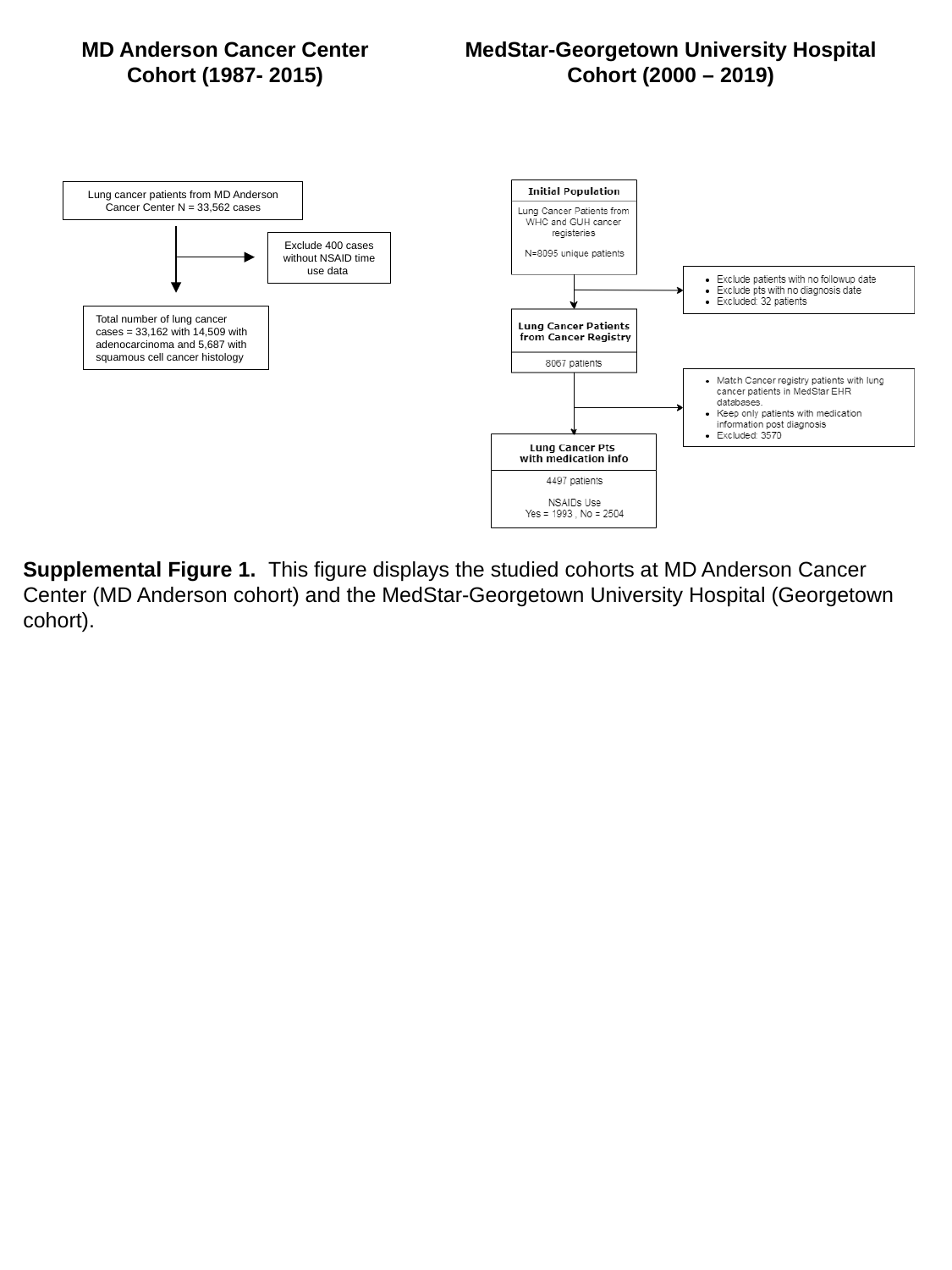

MedStar-Georgetown University Hospital
Cohort (2000 – 2019)
MD Anderson Cancer Center
Cohort (1987- 2015)
Lung cancer patients from MD Anderson Cancer Center N = 33,562 cases
Exclude 400 cases without NSAID time use data
Total number of lung cancer cases = 33,162 with 14,509 with adenocarcinoma and 5,687 with squamous cell cancer histology
Supplemental Figure 1. This figure displays the studied cohorts at MD Anderson Cancer
Center (MD Anderson cohort) and the MedStar-Georgetown University Hospital (Georgetown
cohort).
