# Supplementary material for: Real-world Studies Link NSAID Use to Improved Overall Lung Cancer Survival
Source: Cancer Res Commun. 2022 Jul 6;2(7):590–601. doi: 10.1158/2767-9764.CRC-22-0179 (PMC9273107; doi:10.1158/2767-9764.CRC-22-0179)
Supplement: Supplementary Figure S2 — Supplemental Figure 2. The Kaplan-Meier analysis of overall survival and NSAID use in lung cancer cases within the MedStar-Georgetown University database (Georgetown cohort). [file crc-22-0179-s02.pptx]

## Slide 1
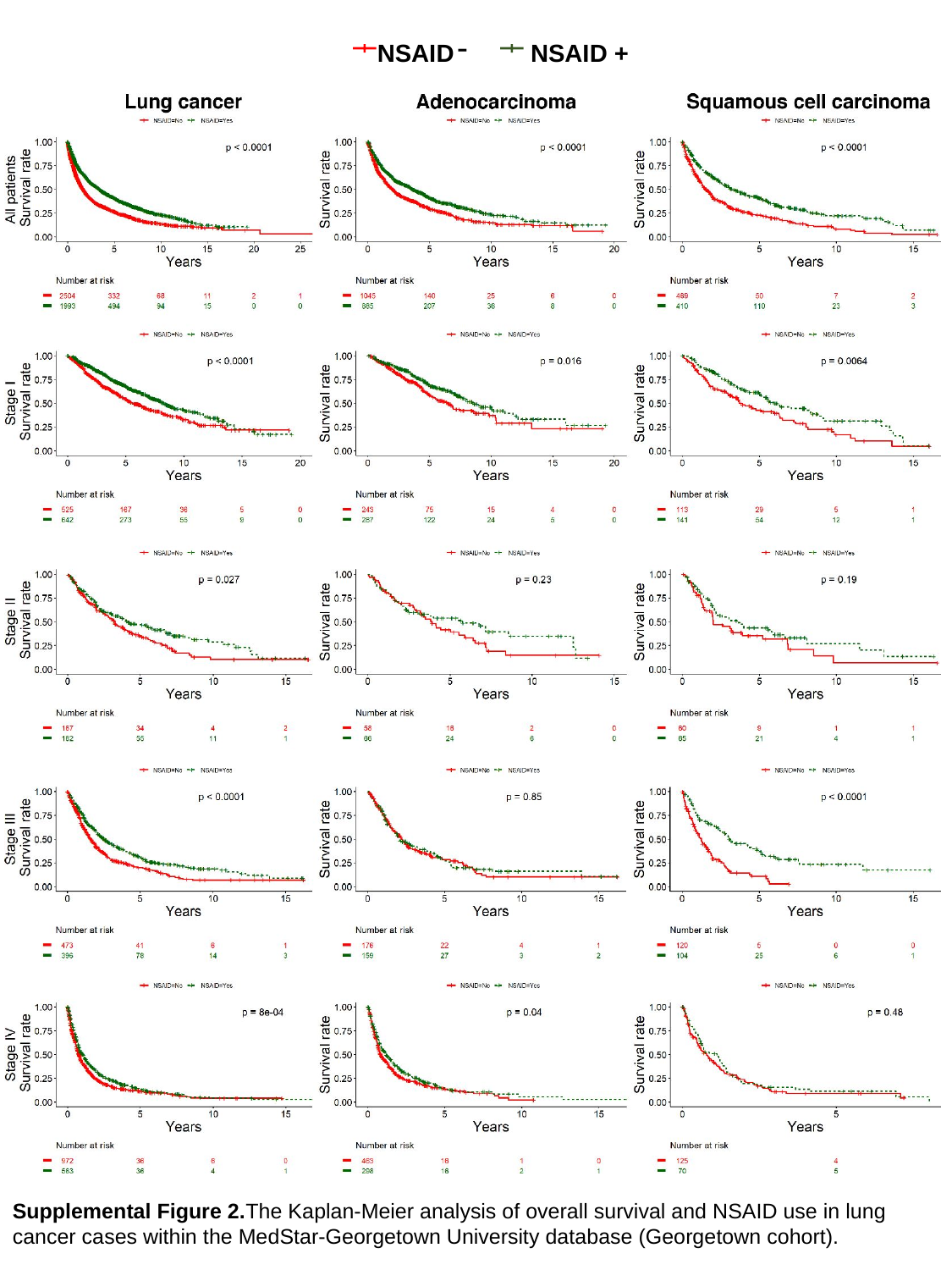

-
NSAID NSAID +
Supplemental Figure 2.The Kaplan-Meier analysis of overall survival and NSAID use in lung cancer cases within the MedStar-Georgetown University database (Georgetown cohort).
