# Supplementary material for: Real-world Studies Link NSAID Use to Improved Overall Lung Cancer Survival
Source: Cancer Res Commun. 2022 Jul 6;2(7):590–601. doi: 10.1158/2767-9764.CRC-22-0179 (PMC9273107; doi:10.1158/2767-9764.CRC-22-0179)
Supplement: Supplementary Figure S3 — Supplemental Figure 3. Landmark analysis of the MD Anderson Cancer Center lung cancer cases. [file crc-22-0179-s03.pptx]

## Slide 1
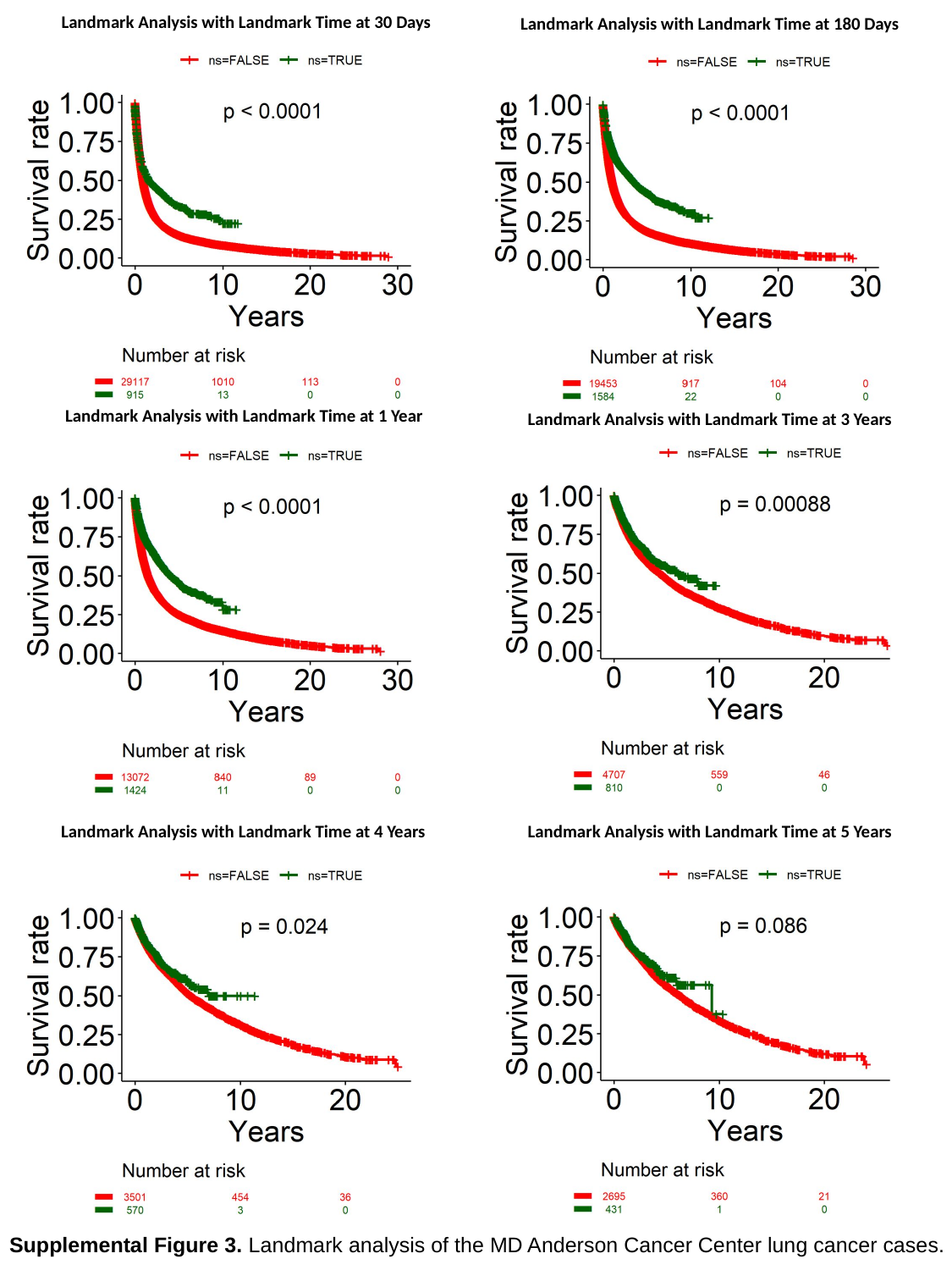

Landmark Analysis with Landmark Time at 30 Days
Landmark Analysis with Landmark Time at 180 Days
Landmark Analysis with Landmark Time at 1 Year
Landmark Analysis with Landmark Time at 3 Years
Landmark Analysis with Landmark Time at 4 Years
Landmark Analysis with Landmark Time at 5 Years
Supplemental Figure 3. Landmark analysis of the MD Anderson Cancer Center lung cancer cases.
