# Supplementary material for: Real-world Studies Link NSAID Use to Improved Overall Lung Cancer Survival
Source: Cancer Res Commun. 2022 Jul 6;2(7):590–601. doi: 10.1158/2767-9764.CRC-22-0179 (PMC9273107; doi:10.1158/2767-9764.CRC-22-0179)
Supplement: Supplementary Figure S4 — Supplemental Figure 4. Landmark analysis of the MedStar-Georgetown University Hospital Database (Georgetown cohort). [file crc-22-0179-s04.pptx]

## Slide 1
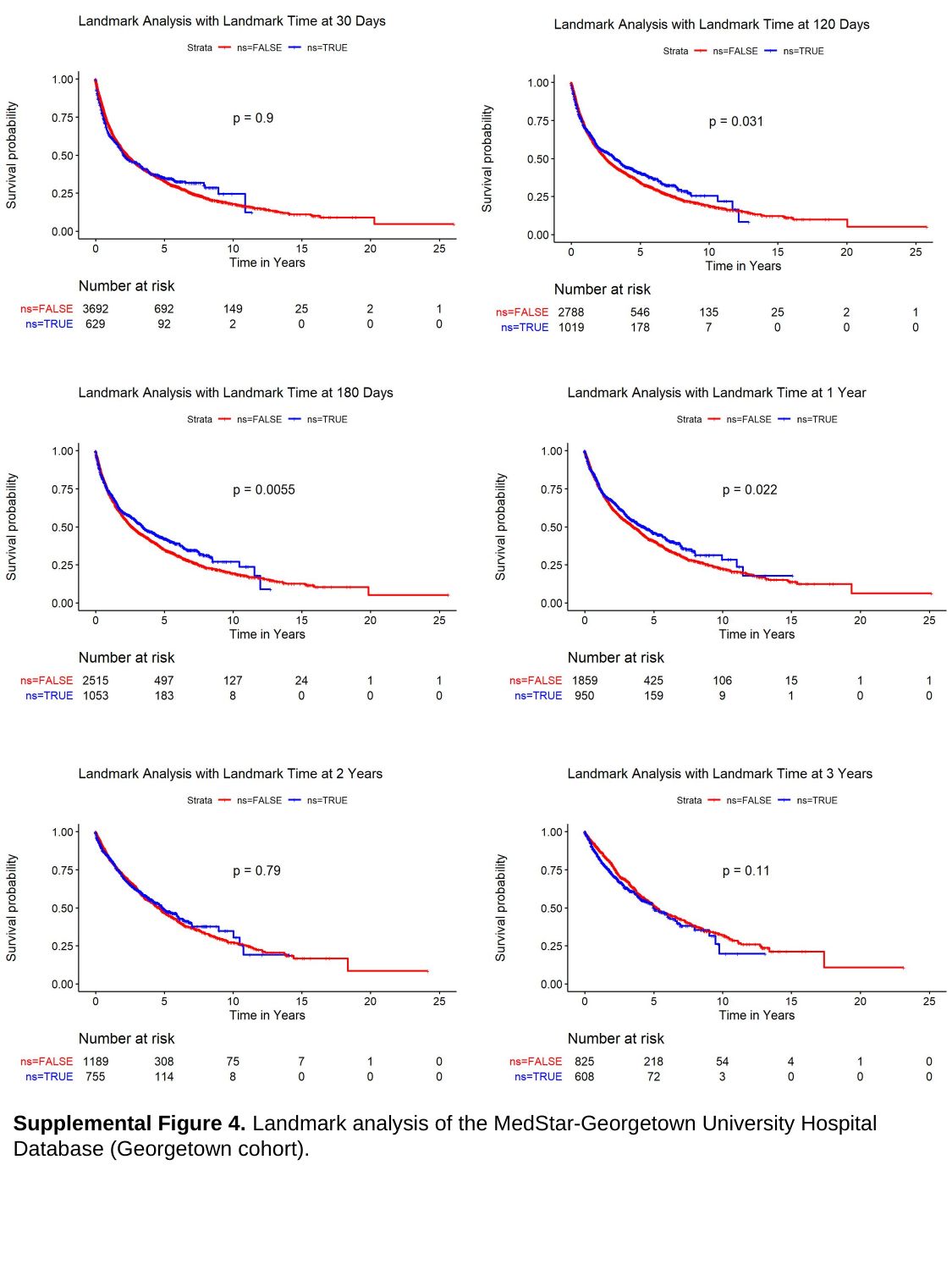

Supplemental Figure 4. Landmark analysis of the MedStar-Georgetown University Hospital
Database (Georgetown cohort).
