# Supplementary material for: Real-world Studies Link NSAID Use to Improved Overall Lung Cancer Survival
Source: Cancer Res Commun. 2022 Jul 6;2(7):590–601. doi: 10.1158/2767-9764.CRC-22-0179 (PMC9273107; doi:10.1158/2767-9764.CRC-22-0179)
Supplement: Supplementary Figure S5 — Supplemental Figure 5. The Kaplan-Meier analysis of overall lung cancer survival by NSAID type in (A) the MD Anderson Cancer Center database (MD Anderson cohort) and (B) the Georgetown cohort. Patients who used multiple NSAID types were excluded from this analysis. [file crc-22-0179-s05.pptx]

## Slide 1
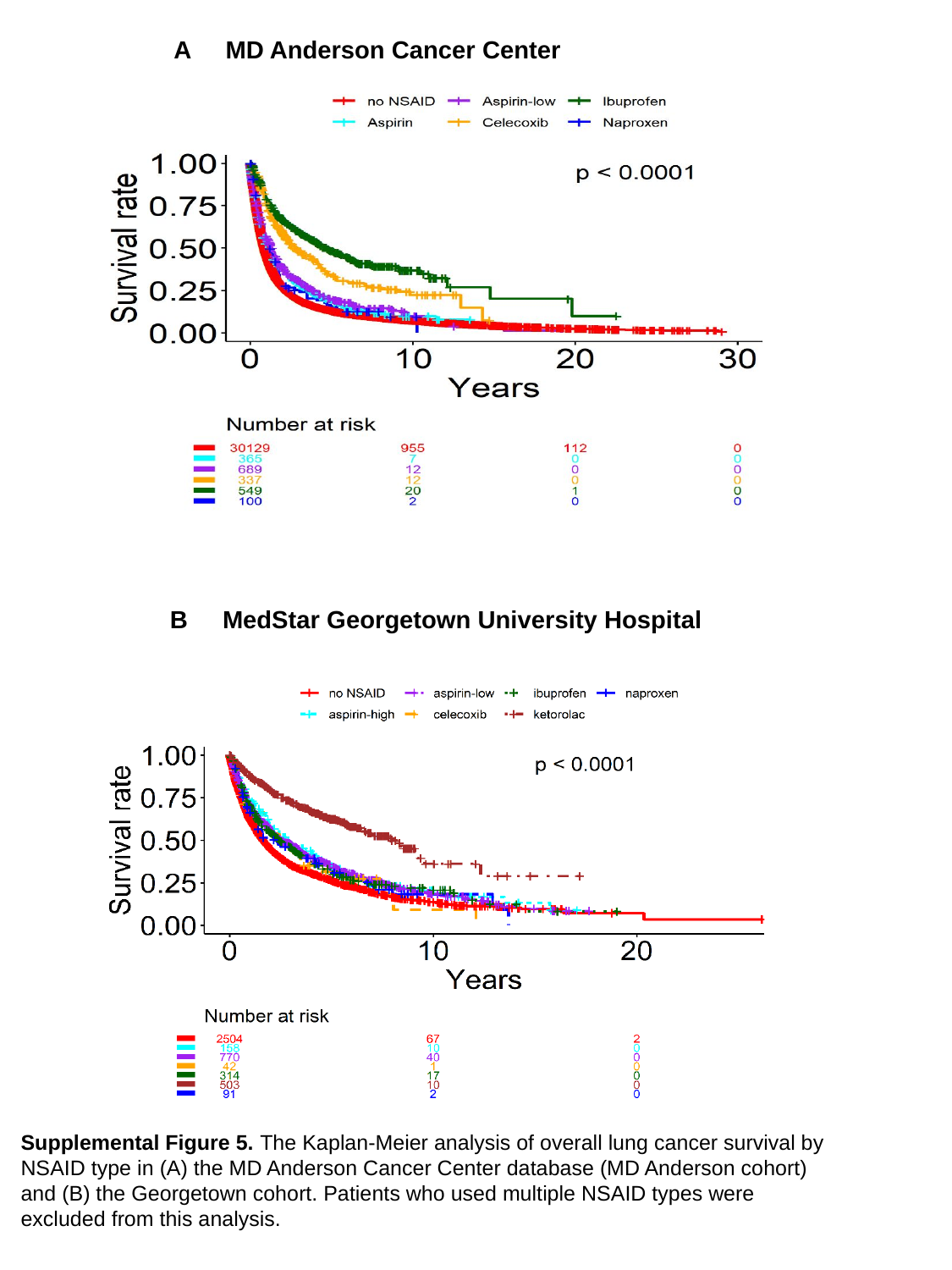

A MD Anderson Cancer Center
B MedStar Georgetown University Hospital
Supplemental Figure 5. The Kaplan-Meier analysis of overall lung cancer survival by NSAID type in (A) the MD Anderson Cancer Center database (MD Anderson cohort) and (B) the Georgetown cohort. Patients who used multiple NSAID types were excluded from this analysis.
