# Supplementary material for: Real-world Studies Link NSAID Use to Improved Overall Lung Cancer Survival
Source: Cancer Res Commun. 2022 Jul 6;2(7):590–601. doi: 10.1158/2767-9764.CRC-22-0179 (PMC9273107; doi:10.1158/2767-9764.CRC-22-0179)
Supplement: Supplementary Table S1 — Supplemental Table 1. Nonsteroidal anti-inflammatory drugs analyzed by Natural Language Processing (NLP). [file crc-22-0179-s06.docx]

| Advil |
| --- |
| Aleve |
| Amlodine |
| Anacin |
| Arthrotec |
| Aspirin |
| Aspirin Low Dose |
| Aspirin/dipyridamole (Aggrenox) |
| Aspirin-Caffeine-Butalbital |
| Aspirin-Dipyridamole |
| Butalbital/aspirin |
| Celebrex |
| Celecoxib |
| Diclofenac Potassium |
| Diclofenac Sodium |
| Ecotrin |
| Etodolac |
| Excedrin |
| Feldene |
| Flurbiprofen |
| Hydrocodone/Ibuprofen (Vicoprofen) |
| Ibuprofen |
| Indocin |
| Indomethacin |
| Ketoprofen |
| Meloxicam (Mobic) |
| Motrin |
| Nabumetone |
| Naprosyn |
| Naproxen |
| Oxaprozin |
| Piroxicam |
| Relafen |
| Sulindac |
| Tolmetin Sodium |
| Voltaren |

**Supplemental Table 1.** Nonsteroidal anti-inflammatory drugs analyzed by Natural Language Processing (NLP).
