# Supplementary material for: Real-world Studies Link NSAID Use to Improved Overall Lung Cancer Survival
Source: Cancer Res Commun. 2022 Jul 6;2(7):590–601. doi: 10.1158/2767-9764.CRC-22-0179 (PMC9273107; doi:10.1158/2767-9764.CRC-22-0179)
Supplement: Supplementary Table S2 — Supplemental Table 2. Lung cancer patient characteristics in the MedStar-Georgetown University cohort. All lung cancers and those with adenocarcinoma (AD) or squamous cell cancer (SCC) histopathology are shown. [file crc-22-0179-s07.docx]

|  | **Lung cancer (n=4,497)** | **AD (n=1,930)** | **SCC (n=879)** |
| --- | --- | --- | --- |
| **Age, median years** | 67 | 66 | 69 |
| **Gender, n (%)** |  |  |  |
| **Female** | 2413 (54%) | 1119 (58%) | 362 (41%) |
| **Male** | 2084 (46%) | 811 (42%) | 517 (59%) |
| **Race, n (%)** |  |  |  |
| **Caucasian/Non-Hispanic White** | 1648 (37%) | 686 (36%) | 322 (37%) |
| **African American/Black** | 2290 (51%) | 976 (51%) | 467 (53%) |
| **American Indian/Alaska Native** | 13 (0.30%) | 6 (0.31%) | 2 (0.23%) |
| **Asian** | 178 (4%) | 97 (5%) | 33 (3.8%) |
| **Hispanic** | 8 (0.2%) | 5 (0.26%) | 0 (0%) |
| **Native Hawaiian/Pacific Islander** | 18 (0.4%) | 10 (0.52%) | 3 (0.34%) |
| **Multi-Racial** | 3 (0.06%) | 2 (0.10%) | 0 (0%) |
| **Other** | 110 (2.4%) | 52 (2.7%) | 13 (1.5%) |
| **Unknown** | 229 (5%) | 96 (5%) | 39 (4.4%) |
| **Smoking status, n (%)** |  |  |  |
| **Non-smoker** | 394 (8.8%) | 226 (12%) | 27 (3.1%) |
| **Former smoker** | 1485 (33%) | 681 (35%) | 305 (35%) |
| **Current smoker** | 125 (2.8%) | 48 (2.5%) | 35 (4%) |
| **NSAID use, n (%)** |  |  |  |
| **No** | 2504 (56%) | 1045 (54%) | 469 (53%) |
| **Yes** | 1993 (44%) | 885 (46%) | 410 (47%) |
|  |  |  |  |
|  |  |  |  |

**Supplemental Table 2.** Lung cancer patient characteristics in the MedStar-Georgetown University cohort. All lung cancers and those with adenocarcinoma (AD) or squamous cell cancer (SCC) histopathology are shown.
