# Supplementary material for: Real-world Studies Link NSAID Use to Improved Overall Lung Cancer Survival
Source: Cancer Res Commun. 2022 Jul 6;2(7):590–601. doi: 10.1158/2767-9764.CRC-22-0179 (PMC9273107; doi:10.1158/2767-9764.CRC-22-0179)
Supplement: Supplementary Table S3 — Supplemental Table 3. Multicovariable Cox proportional hazards model with variables associated with overall survival for the MD Anderson Cancer Center cohort. [file crc-22-0179-s08.docx]

|  | **Lung cancer** | | | **Adenocarcinoma** | | | **Squamous cell carcinoma** | | |
| --- | --- | --- | --- | --- | --- | --- | --- | --- | --- |
| **Characteristics** | **HR***^1^* | **95% CI***^1^* | **P-value** | **HR***^1^* | **95% CI***^1^* | **P-value** | **HR***^1^* | **95% CI***^1^* | **P-value** |
| **Age** | 1.00 | 1.00, 1.01 | <0.001 | 1.00 | 1.00, 1.00 | 0.6 | 1.01 | 1.00, 1.01 | 0.007 |
| **Gender** |  |  |  |  |  |  |  |  |  |
| **Female** | — | — |  | — | — |  | — | — |  |
| **Male** | 1.23 | 1.19, 1.27 | <0.001 | 1.26 | 1.20, 1.32 | <0.001 | 1.12 | 1.04, 1.21 | 0.004 |
| **Race** |  |  |  |  |  |  |  |  |  |
| **Caucasian/Non-Hispanic White** | — | — |  | — | — |  | — | — |  |
| **African American/Black** | 1.20 | 1.14, 1.27 | <0.001 | 1.20 | 1.10, 1.30 | <0.001 | 1.21 | 1.07, 1.36 | 0.002 |
| **Hispanic** | 1.01 | 0.94, 1.09 | 0.7 | 1.02 | 0.92, 1.13 | 0.7 | 1.03 | 0.86, 1.24 | 0.7 |
| **Others** | 0.90 | 0.82, 0.98 | 0.017 | 0.84 | 0.75, 0.94 | 0.002 | 1.19 | 0.91, 1.55 | 0.2 |
| **Smoking status** |  |  |  |  |  |  |  |  |  |
| **Never smoker** | — | — |  | — | — |  | — | — |  |
| **Former smoker** | 1.20 | 1.15, 1.26 | <0.001 | 1.14 | 1.07, 1.21 | <0.001 | 0.86 | 0.76, 0.98 | 0.020 |
| **Current smoker** | 1.28 | 1.22, 1.33 | <0.001 | 1.13 | 1.07, 1.21 | <0.001 | 0.96 | 0.85, 1.09 | 0.6 |
| **NSAID** |  |  |  |  |  |  |  |  |  |
| **no** | — | — |  | — | — |  | — | — |  |
| **yes** | 0.58 | 0.55, 0.61 | <0.001 | 0.61 | 0.57, 0.66 | <0.001 | 0.61 | 0.55, 0.68 | <0.001 |
| *^1^*HR = Hazard Ratio, CI = Confidence Interval | | | | | | | | | |

**Supplemental Table 3**. Multicovariable Cox proportional hazards model with variables associated with overall survival for the MD Anderson Cancer Center cohort.
