# Supplementary material for: Real-world Studies Link NSAID Use to Improved Overall Lung Cancer Survival
Source: Cancer Res Commun. 2022 Jul 6;2(7):590–601. doi: 10.1158/2767-9764.CRC-22-0179 (PMC9273107; doi:10.1158/2767-9764.CRC-22-0179)
Supplement: Supplementary Table S4 — Supplemental Table 4. Multicovariable analysis with variables associated with overall survival for the Georgetown cohort. [file crc-22-0179-s09.docx]

|  | **Lung cancer** | | | **Adenocarcinoma** | | | **Squamous cell carcinoma** | | |
| --- | --- | --- | --- | --- | --- | --- | --- | --- | --- |
| **Characteristics** | **HR***^1^* | **95% CI***^1^* | **P-value** | **HR***^1^* | **95% CI***^1^* | **P-value** | **HR***^1^* | **95% CI***^1^* | **P-value** |
| **Age** | 1.013 | 1.009, 1.016 | 3.33e-12 | 1.010 | 1.004, 1.016 | 4.05e-04 | 1.018 | 1.008, 1.028 | 2.18e-4 |
| **Gender** |  |  |  |  |  |  |  |  |  |
| **Female** | — | — |  | — | — |  | — | — |  |
| **Male** | 1.336 | 1.241, 1.438 | 1.15e-14 | 1.432 | 1.276, 1.608 | 1.10e-09 | 1.211 | 1.024, 1.433 | 0.025 |
| **Race** |  |  |  |  |  |  |  |  |  |
| **Caucasian/Non-Hispanic White** | — | — |  | — | — |  | — | — |  |
| **African American/Black** | 1.414 | 1.305, 1.532 | 3.31e-17 | 1.383 | 1.216, 1.574 | 7.92e-07 | 1.262 | 1.059, 1.504 | 0.009 |
| **American Indian/Alaska Native** | 1.609 | 0.910, 2.847 | 0.102 | 2.131 | 0.943, 4.815 | 0.069 | 4.071 | 1.001,16.559 | 0.05 |
| **Asian** | 0.813 | 0.656, 1.008 | 0.059 | 0.866 | 0.646, 1.162 | 0.338 | 0.731 | 0.456, 1.172 | 0.193 |
| **Hispanic** | 1.119 | 0.418, 2.997 | 0.823 | 1.501 | 0.557, 4.050 | 0.422 | N/A | N/A | N/A |
| **Native Hawaiian/Pacific Islander** | 0.737 | 0.367, 1.480 | 0.391 | 1.182 | 0.489, 2.859 | 0.711 | 0.535 | 0.075, 3.838 | 0.534 |
| **Other (includes 3 multi-racial individuals)** | 1.456 | 1.150, 1.843 | 0.005 | 1.130 | 0.780, 1.638 | 0.517 | 1.535 | 0.810, 2.908 | 0.189 |
| **Unknown** | 1.491 | 1.254, 1.773 | 6.14e-06 | 1.295 | 0.982, 1.708 | 0.067 | 1.530 | 1.007, 2.325 | 0.046 |
| **Smoking status** |  |  |  |  |  |  |  |  |  |
| **Never smoker** | — | — |  | — | — |  | — | — |  |
| **Former smoker** | 1.152 | 0.994, 1.336 | 0.06 | 1.086 | 0.884, 1.334 | 0.433 | 0.713 | 0.447, 1.138 | 0.156 |
| **Current smoker** | 0.998 | 0.745, 1.337 | 0.99 | 0.766 | 0.476, 1.230 | 0.270 | 0.542 | 0.267, 1.098 | 0.088 |
| **Unknown** | 1.342 | 1.164, 1.548 | 5.22e-05 | 1.321 | 1.083, 1.612 | 0.007 | 0.843 | 0.534, 1.332 | 0.465 |
| **NSAID use** |  |  |  |  |  |  |  |  |  |
| **No** | — | — |  | — | — |  | — | — |  |
| **Yes** | 0.680 | 0.631, 0.732 | 4.08e-24 | 0.734 | 0.654, 0.824 | 1.78e-07 | 0.613 | 0.519, 0.725 | 8.75e-09 |

*^1^*HR = Hazard Ratio, CI = Confidence Interval

**Supplemental Table 4.** Multicovariable analysis with variables associated with overall survival for the Georgetown cohort.
