# Supplementary material for: Real-world Studies Link NSAID Use to Improved Overall Lung Cancer Survival
Source: Cancer Res Commun. 2022 Jul 6;2(7):590–601. doi: 10.1158/2767-9764.CRC-22-0179 (PMC9273107; doi:10.1158/2767-9764.CRC-22-0179)
Supplement: Supplementary Table S5 — Supplementary Table 5: The MD Anderson and Georgetown cohorts 5-year survival rates and differences of 5-year restricted mean survival time in months between NSAID users and non-users for all patients and by stage and histopathology corresponding to Figure 1. The abbreviations are AD (adenocarcinoma) and SCC (squamous cell cancer), respectively. [file crc-22-0179-s10.docx]

| \|  \|  \| **MD Anderson Cohort** \| \| \| **Georgetown Cohort** \| \| \| \| --- \| --- \| --- \| --- \| --- \| --- \| --- \| --- \| \|  \|  \| **Lung cancer** \| **AD** \| **SCC** \| **Lung cancer** \| **AD** \| **SCC** \| \| **All patients** \| 5-yr survival  rate for NSAID users \| 29.7% \| 27.1% \| 31.3% \| 40.5% \| 40.5% \| 40.7% \| \|  \| 5-yr survival  rate for NSAID non-users \| 13.1% \| 12.9% \| 14.4% \| 26.5% \| 29.2% \| 23.0% \| \|  \| difference in 5-year RMST  (months) \| 11.6 \| 10.9 \| 11.0 \| 8.6 \| 6.7 \| 10.4 \| \| **Stage I** \| 5-yr survival  rate for NSAID users \| 70.7% \| 68.7% \| 63.3% \| 67.2% \| 67.8% \| 58.8% \| \|  \| 5-yr survival  rate for NSAID non-users \| 58.0% \| 61.4% \| 47.3% \| 53.8% \| 57.5% \| 42.8% \| \|  \| difference in 5-year RMST  (months) \| 5.9 \| 3.9 \| 6.6 \| 5.5 \| 3.3 \| 7.4 \| \| **Stage II** \| 5-yr survival  rate for NSAID users \| 49.9% \| 45.2% \| 52.6% \| 46.9% \| 53.9% \| 43.6% \| \|  \| 5-yr survival  rate for NSAID non-users \| 38.0% \| 43.8% \| 31.3% \| 34.4 % \| 38.8% \| 35.7% \| \|  \| difference in 5-year RMST  (months) \| 7.1 \| 5.0 \| 8.3 \| 3.5 \| 1.0 \| 4.9 \| \| **Stage III** \| 5-yr survival  rate for NSAID users \| 30.5% \| 29.8% \| 29.0% \| 30.1% \| 27.0% \| 36.9% \| \|  \| 5-yr survival  rate for NSAID non-users \| 17.5% \| 19.4% \| 15.4% \| 19.9% \| 28.1% \| 11.0% \| \|  \| difference in 5-year RMST  (months) \| 9.3 \| 9.1 \| 7.7 \| 7.3 \| 0.6 \| 16.1 \| \| **Stage IV** \| 5-yr survival  rate for NSAID users \| 7.2% \| 10.1% \| 2.0% \| 13.4% \| 13.1% \| 11.2% \| \|  \| 5-yr survival  rate for NSAID non-users \| 4.2% \| 5.2% \| 2.6% \| 11.4% \| 13.7% \| 9.2% \| \|  \| difference in 5-year RMST  (months) \| 3.6 \| 4.1 \| 1.1 \| 3.8 \| 3.1 \| 2.0 \|   **Supplementary Table 5:** The MD Anderson and Georgetown cohorts 5-year survival rates and differences of 5-year restricted mean survival time in months between NSAID users and non-users for all patients and by stage and histopathology corresponding to **Figure 1.** The abbreviations are AD (adenocarcinoma) and SCC (squamous cell cancer), respectively. |
| --- | --- | --- | --- | --- | --- | --- | --- | --- | --- | --- | --- | --- | --- | --- | --- | --- | --- | --- | --- | --- | --- | --- | --- | --- | --- | --- | --- | --- | --- | --- | --- | --- | --- | --- | --- | --- | --- | --- | --- | --- | --- | --- | --- | --- | --- | --- | --- | --- | --- | --- | --- | --- | --- | --- | --- | --- | --- | --- | --- | --- | --- | --- | --- | --- | --- | --- | --- | --- | --- | --- | --- | --- | --- | --- | --- | --- | --- | --- | --- | --- | --- | --- | --- | --- | --- | --- | --- | --- | --- | --- | --- | --- | --- | --- | --- | --- | --- | --- | --- | --- | --- | --- | --- | --- | --- | --- | --- | --- | --- | --- | --- | --- | --- | --- | --- | --- | --- | --- | --- | --- | --- | --- | --- | --- | --- | --- | --- | --- | --- | --- | --- | --- | --- | --- | --- | --- |
