# Supplementary material for: Real-world Studies Link NSAID Use to Improved Overall Lung Cancer Survival
Source: Cancer Res Commun. 2022 Jul 6;2(7):590–601. doi: 10.1158/2767-9764.CRC-22-0179 (PMC9273107; doi:10.1158/2767-9764.CRC-22-0179)
Supplement: Supplementary Table S6 — Supplementary Table 6: The MD Anderson cohort 5-year survival rate and difference of 5-year restricted mean survival time in months between NSAID users and non-users by gender, race, and smoking status corresponding to Figure 2. Comparisons are made to the Georgetown cohort. [file crc-22-0179-s11.docx]

| \|  \|  \|  \| **MD Anderson Cohort** \| **Georgetown Cohort** \| \| --- \| --- \| --- \| --- \| --- \| \| **Gender** \| **Male** \| 5-yr survival rate  for NSAID users \| 25.0% \| 33.4% \| \|  \|  \| 5-yr survival rate for NSAID non-users \| 10.6% \| 21.5% \| \|  \|  \| difference in 5-year RMST (months) \| 10.8 \| 8.2 \| \|  \| **Female** \| 5-yr survival rate  for NSAID users \| 35.2% \| 46.3% \| \|  \|  \| 5-yr survival rate for NSAID non-users \| 16.1% \| 30.8% \| \|  \|  \| difference in 5-year RMST (months) \| 12.5 \| 8.8 \| \| **Race** \| **African American/Black** \| 5-yr survival rate  for NSAID users \| 22.2% \| 36.5% \| \|  \|  \| 5-yr survival rate for NSAID non-users \| 10.5% \| 22.0% \| \|  \|  \| difference in 5-year RMST (months) \| 9.7 \| 9.2 \| \|  \| **Caucasian/Non-Hispanic White** \| 5-yr survival rate  for NSAID users \| 30.8% \| 45.2% \| \|  \|  \| 5-yr survival rate for NSAID non-users \| 13.2% \| 31.9% \| \|  \|  \| difference in 5-year RMST (months) \| 12.2 \| 7.0 \| \|  \| **Hispanic** \| 5-yr survival rate  for NSAID users \| 27.4% \| 49.4% \| \|  \|  \| 5-yr survival rate for NSAID non-users \| 14.6% \| 36.4% \| \|  \|  \| difference in 5-year RMST (months) \| 9.3 \| 5.6 \| \| **Smoking Status** \| **Never smoker** \| 5-yr survival rate  for NSAID users \| 39.0% \| 39.9% \| \|  \|  \| 5-yr survival rate for NSAID non-users \| 18.9% \| 29.1% \| \|  \|  \| difference in 5-year RMST (months) \| 11.6 \| 8.1 \| \|  \| **Former smoker** \| 5-yr survival rate  for NSAID users \| 27.2% \| 62.8% \| \|  \|  \| 5-yr survival rate for NSAID non-users \| 14.6% \| 31.5% \| \|  \|  \| difference in 5-year RMST (months) \| 10.0 \| 11.9 \| \|  \| **Current smoker** \| 5-yr survival rate  for NSAID users \| 30.0% \| 33.4% \| \|  \|  \| 5-yr survival rate for NSAID non-users \| 13.1% \| 21.5% \| \|  \|  \| difference in 5-year RMST (month) \| 12.1 \| 8.2 \|   **Supplementary Table 6**: The MD Anderson cohort 5-year survival rate and difference of 5-year restricted mean survival time in months between NSAID users and non-users by gender, race, and smoking status corresponding to **Figure 2**. Comparisons are made to the Georgetown cohort. |
| --- | --- | --- | --- | --- | --- | --- | --- | --- | --- | --- | --- | --- | --- | --- | --- | --- | --- | --- | --- | --- | --- | --- | --- | --- | --- | --- | --- | --- | --- | --- | --- | --- | --- | --- | --- | --- | --- | --- | --- | --- | --- | --- | --- | --- | --- | --- | --- | --- | --- | --- | --- | --- | --- | --- | --- | --- | --- | --- | --- | --- | --- | --- | --- | --- | --- | --- | --- | --- | --- | --- | --- | --- | --- | --- | --- | --- | --- | --- | --- | --- | --- | --- | --- | --- | --- | --- | --- | --- | --- | --- | --- | --- | --- | --- | --- | --- | --- | --- | --- | --- | --- | --- | --- | --- | --- | --- | --- | --- | --- | --- | --- | --- | --- | --- | --- | --- | --- | --- | --- | --- | --- | --- | --- | --- | --- |
